# Ultrasound versus conventional chest radiography after ultrasound guided insertion of a central line

**DOI:** 10.1186/2197-425X-3-S1-A606

**Published:** 2015-10-01

**Authors:** MJ Blans, FH Bosch

**Affiliations:** Intensive Care, Rijnstate Hospital, Arnhem, Netherlands; Rijnstate Hospital, Arnhem, Netherlands

## Introduction

Central venous catheters (CVC's) are used frequently in modern medicine. After CVC insertion a conventional chest radiograph (CCR) is often ordered to detect complications and/ position There are reports that the use of ultrasound (US) might be better in detecting complications and correct or incorrect position and is more time efficient.

## Objectives

We conducted a prospective observational study in which we compared the use of US versus conventional chest radiograph (CCR) in patients receiving a CVC for the detection of post insertion complications and to confirm proper CVC position.

## Methods

Adult patients in need for a CVC could be included. All CVC´s were inserted under direct in plane US guidance. After insertion US was used to screen for correct or incorrect CVC position (in the case of a CVC in the Internal Jugular Vein (IJV) the ipsilateral subclavian vein (SCV) and in case of a CVC in the SCV the ipsilateral IJV was examined. On the ipsilateral side the two upper "blue points" were examined to rule out a pneumothorax. Finally cardiac ultrasound (CUS) was used to chech correct or incorrect CVC position in the heart or inferior cava vein and with the use of 5 cc agitated saline the pattern of microbubbles in the right atrium was recorded to confrim proper position. The time needed to obtain a CCR was noted. The results of the CCR were compared to the US results in a 2 x 2 table (US correct and incorrect position versus CCR correct and incorrect position).

## Results

46 Patients were included: 3 SCV and 43 IJV CVC´s were inserted. The results of US and CCR in detecting pneumothorax were equal (n = 0). In 1 patient no CUS view could be obtained, in the other 45 the results of CCR and US were the same (correct position of the CVC). The sensitivity of US for detecting propper CVC position was 1,0 (0,9-1.0).

The mean time needed waiting for the result of the CCR was 20 (0-195) minutes. we excluded the waiting time for CCR in 1 patient during a hospital system faliure for 2 days.

## Conclusions

US is comparable to CCR in detecting proper position and complications after CVC insertion and is more time efficient. If a mode of control after CVC insertion is wanted, US is the preferred method.
